# Documentation of cognitive impairment screening amongst older hospitalised Australians: a prospective clinical record audit

**DOI:** 10.1186/s12877-023-04394-z

**Published:** 2023-10-18

**Authors:** Radhika Rice, Jamie Bryant, Rob Sanson Fisher

**Affiliations:** 1 Hunter New England Local Health District, Newcastle, NSW Australia; 2 School of Medicine and Public Health, College of Health, Medicine and Wellbeing, University of Newcastle, Callaghan, NSW Australia; 3 Equity in Health and Wellbeing program, Hunter Medical Research Institute, New Lambton Heights, NSW Australia

**Keywords:** Cognitive impairment, Cognitive dysfunction, Cognitive screening, Older people

## Abstract

**Background:**

Failure to detect cognitive impairment (CI) in hospitalised older inpatients has serious medical and legal implications, including for the implementation of care planning. This mixed methods study aimed to determine amongst hospital in-patients aged ≥ 65 years: (1) Rates of documentation of screening for CI, including the factors associated with completion of screening; (2) Rates of undocumented CI amongst patients who had not received screening during their admission; (3) Healthcare provider practices and barriers related to CI screening.

**Methods:**

A mixed methods study incorporating a clinical audit and interviews with healthcare providers was conducted at one Australian public hospital. Patients were eligible for inclusion if they were aged 65 years and older and were admitted to a participating ward for a minimum of 48 h. Patient characteristics, whether CI screening had been documented, were extracted using a template. Patients who had not been screened for CI completed the Montreal Cognitive Assessment (MoCA) to determine cognitive status. Interviews were conducted with healthcare providers to understand practices and barriers to screening for CI.

**Results:**

Of the 165 patients included, 34.5% (n = 57) had screening for CI documented for their current admission. Patients aged > 85 years and those with two or more admissions had greater odds of having CI screening documented. Among patients without CI screening documented, 72% (n = 78) were identified as cognitively impaired. While healthcare providers agreed CI screening was beneficial, they identified lack of time and poor knowledge as barriers to undertaking screening.

**Conclusions:**

CI is frequently unrecognised in the hospital setting which is a missed opportunity for the provision of appropriate care. Future research should identify feasible and effective strategies to increase implementation of CI screening in hospitals.

**Supplementary Information:**

The online version contains supplementary material available at 10.1186/s12877-023-04394-z.

## Background

Whilst older adults aged 65 years and over comprise just 16% of the Australian population [1] they are overrepresented as hospital inpatients. In 2020, adults aged over 65 years accounted for 40% of all hospital admissions and 41% of all overnight hospitalisations [2]. Current projections estimate the number of older Australians will more than double by 2066 [3]. This will have significant impacts on the care provided in hospitals, and the healthcare system more broadly.

Cognitive impairment (CI) refers to changes in cognition with deficits in one or more domains of memory, language, executive function, attention, perceptual-motor, and social cognition. CI can range from age related cognitive decline, to mild cognitive impairment and dementia. In complex acute-care settings proper diagnosis of dementia is challenging due to the presence of other causes of cognitive dysfunction (e.g., delirium, head injury), and full diagnostics for assessment may not be feasible or appropriate. Hence the term CI is adopted to broadly describe cognitive dysfunction in the acute setting without the requirement to ascribe a diagnosis [4]. In Australia, it is estimated that 29-38% of older patients admitted to hospital have CI [5, 6] and 25% aged over 85 years have dementia [7].

Patients admitted to hospital with CI have higher rates of hospital morbidity and mortality; higher incidence of hospital acquired complications including hospital acquired infections, and pressure injuries; longer hospital stays; greater functional decline; and higher rates of discharge into residential aged care than patients without CI [6–12]. Failure to identify CI in hospital can lead to insufficient or inappropriate care including assumed cognitive capability to understand complex medication regimens and not implementing delirium preventative strategies. Early and timely detection of CI in older inpatients therefore has the potential to both reduce adverse events during hospitalisation (i.e. delirium, falls) and provide opportunities to engage patients and their support persons in advanced care planning to promote autonomy and control over future health care decisions [13–15].

Because of the known benefits of detecting CI amongst older inpatients, routine screening for CI is recommended. The Australian Commission on Safety and Quality in Healthcare’s National Safety and Quality Health Service Standards for Delirium [16] and Guide for providing care for patients with CI at risk of delirium [17] specify that screening for CI should be embedded into routine clinical care for patients aged *≥* 65 years (or *≥* 45 years for Aboriginal and Torres Strait Islander people). This is reflected in local hospital guidelines. For example, the Hunter New England Local Health District policies ‘*Screening, Assessment and Management of Delirium in Adults’* [18] and ‘*Care of Cognitively impaired Older* people’ [19] specify that all patients aged ≥ 65 (≥ 45 for Aboriginal and Torres Strait Islander peoples) require a cognitive screen within 24 hours of admission using either the 4AT, Abbreviate Mental Test Score (AMTS), the Six Item Screener or the Rowland Universal Dementia Scale (RUDAS). While policies recommend that CI screening should be part of routine care [16, 17], previous studies show this rarely occurs in routine practice [6, 20].

A national evaluation in four hospitals across Australia [6] estimated that while 40% of older adults who receive screening using a validated tool are found to have CI, screening is conducted less than 60% of the time in hospital [6]. A range of factors that impede screening for CI have been identified. At the healthcare provider level, barriers include limited time within existing workloads, onerous tools, lack of confidence in screening, limited human resources, on over reliance on clinical knowledge/skills, a reluctance to discuss a sensitive topic, and lack of access of specialists to refer to if CI is identified [4, 21–23]. Patient level factors include not being able to participate in cognitive screening (e.g., aphasia) and a fear of the outcomes of screening [4, 21–23]. Understanding the barriers to screening for CI is important to guide and inform interventions to improve rates of screening. To date, no studies have comprehensively examined the prevalence of screening for CI amongst older hospitalised inpatients, while also quantifying rates of undocumented CI and practices and barriers to screening amongst health professionals in the same setting.

This mixed methods study with a prospective clinical record audit for quantitative data and healthcare interviews for qualitative data aimed to:

1. Determine amongst hospital in-patients aged ≥ 65 years:

- rates of documentation of screening for CI, including the factors associated with completion of screening for CI.
- rates of undocumented CI amongst patients who had not received screening as part of their current admission.

2. Understand healthcare provider perceptions of current practices and barriers to screening for CI in the hospital setting.

## Methods

### Design

A mixed methods study that included a prospective audit of clinical records of geriatric inpatients and interviews with healthcare providers was conducted. The interviews were performed to provide complementary perspectives on current practices and barriers to screening for CI in real world clinical practice. The COREQ checklist was used in reporting qualitative study findings [24].

### Setting

Medical inpatient wards at a Public acute group B hospital (AIHW classification) in the Hunter New England Local Health District were eligible for inclusion in the clinical audit. The Hunter New England Local Health District provides services to 12% of the NSW population with over 2.7 million patients supported each year and more than 225,000 stays per year [25]. The average length of stay is 3 days with 42% of admissions aged ≥ 65 years. These statistics are consistent with the national average length of stay and age of admissions [2]. The population of patients serviced by the Hunter New England Local Health District is diverse including a mix of metropolitan, regional, rural and remote communities and mirrors the Australian healthcare system [26].

### Clinical record audit

#### Eligibility

Patients were eligible for inclusion in the clinical record audit if they were aged ≥ 65 years; were inpatients admitted to a participating ward; had been admitted for a minimum of 48 h to enable adequate time for completion of assessment; were determined to be physically well enough to participate by the Nurse Unit Manager; and were determined to not be in a severe delirium that limited capacity to consent to the cognitive assessment or impacted their ability to complete the Montreal Cognitive Assessment as assessed by the geriatrics trainee.

#### Recruitment

Inpatients admitted at the participating hospital who met the eligibility criteria as assessed by the geriatrics trainee were selected at random for inclusion in the audit before undertaking data extraction.

#### Data collection

De-identified data was collected from the clinical records of patients using an audit template from February to September 2020. A systematic sampling approach was adopted where the geriatric trainee approached patients in odd numbered beds. This method of sampling was adopted to eliminate bias from over or under sampling close observation bay beds or single infectious rooms in the medical ward that may have been more likely to have patients with cognitive impairment.

#### Measures

The following information was extracted from each record.

*Demographic characteristics.* Age, gender, relationship status, number of admissions in the past 12 months were recorded.

*Clinical information.* The following information was recorded: [1] whether the patient had the following medical conditions (current or active; yes/no): dementia or mild cognitive impairment; cancer; heart conditions; respiratory conditions; chronic kidney conditions; endocrine conditions; nutritional or metabolic disorders; rheumatological conditions; gastrointestinal conditions; neurological conditions; urinary or reproductive conditions; mental health conditions; [2] whether the patient was currently receiving palliative care (yes/no/unknown); and [3] the patient’s estimated Eastern Cooperative Oncology Group (ECOG) performance status. While most commonly used in practice and in research for oncology and patients, ECOG has been used in national multi-centre health record audits including with patients with dementia as a proxy for functional status given it is a relatively simple and quick assessment tool [27, 28, 29]. Following review of the clinical information recorded, the auditor noted the patient’s likely functional status using five defined categories: 0 if fully active, able to carry out all daily living activities; 1 if restricted in physically strenuous activity, but ambulatory and able to carry out work of a light or sedentary nature; 2 if ambulatory and capable of all self-care but unable to carry out any work activities; 3 if capable of only limited self-care, confined to bed or chair more than 50% of waking hours; 4 if completely disabled, unable to carry out any self-care, totally confined to bed or chair. If they were unable to estimate likely degree of disability, they selected ‘insufficient information available’.

*Screening for CI.* The auditor recorded whether the patient had received screening for CI (yes/no) during the admission. If screening had been conducted, they also recorded which tool was used, and if the score indicated CI. CI was indicated by a score of > 4 on the 4AT, > 8 on the 6 Item Screener, and ≤ 6 on the AMTS.

*Assessment of CI.* Patients that did not have screening for CI documented in their clinical record were administered the Montreal Cognitive Assessment (MoCA) by the geriatrics trainee. The MoCA is a validated tool to detect CI that contains 30 questions assessing eight cognitive domains: orientation, short term memory, delayed memory, executive function, visuospatial ability, abstraction, language, and attention. It can be administered in approximately 10 min and is scored out of 30. It has adequate psychometric properties (sensitivity = 83.9%, specificity = 74.6, internal consistency α = 0.78, and test re-test reliability 0.88) [30]. A score of ≤ 25 was considered as indicative of CI. Given it is a concise, validated tool that has a higher sensitivity of picking up mild cognitive impairment, MoCA was chosen over other brief screening tools mentioned in the policy (4AT,6 Item Screener, AMTS or RUDAS) [31–34].

#### Analysis

Statistical analyses were programmed using SAS v9.4 [35]. A priori, statistical significance was set at p-value < 0.05. Descriptive statistics are reported for all relevant variables, including frequencies and percentages for categorical variables, and means, standard deviations, medians, and ranges for continuous variables. A multivariable logistic regression analyses was conducted to examine the factors associated with having screening for CI documented in the clinical record.

### Qualitative interviews

#### Eligibility

Healthcare providers were eligible for participation in interviews if they were medical officers or members of the Aged Care Services in Emergency Team at the participating hospital.

#### Recruitment

An email was sent to all Medical Officers and Aged Care Service Emergency Team nurses at the participating hospital. The email included a detailed study information statement and asked those interested in participating to contact a member of the research team to schedule a convenient time for an interview.

#### Data collection

Interviews focused on current practices for screening for cognitive impairment and perceived barriers to screening. Each interview was guided by a semi-structured interview guide developed with input from geriatricians and health behaviour researchers. The final interview guide (Appendix A) covered the following domains: awareness of hospital policies regarding CI screening, CI screening tools used, frequency of screening, benefits of CI screening and barriers to routine CI screening.

All interviews were conducted face-to-face, at the participating hospital and audio recorded with participant consent by the geriatrics advanced trainee (RR).

#### Analysis

Audio recordings of interviews were transcribed by a professional transcription service. Data were analysed using Nvivo software. An inductive qualitative content analysis approach was chosen. This method was considered particularly suited to gain in-depth insights into participants’ perspectives which are grounded in the actual data, rather than researchers’ preconceived categories and theoretical perspectives [36]. Each whole interview was considered as a unit of analysis. An experienced researcher (JB: PhD) read the transcripts line by line and examined, compared, and categorized their content to apply a paraphrase or label (a “code”) which described what was interpreted in each section as important. Based on the initial codes, more abstract categories were developed [37]. The codes and categories were used to form a coding matrix which was reviewed by all members of the research team. Based on the coding matrix, we generated threads of meaning across categories (i.e. themes) and thus analysed both latent and manifest content derived from the data [38]. The robustness of the conclusions was tested on the basis of each case by comparing codes within each interview, as well as independently of cases by comparing codes between interviews [39].

### Ethics approval

Ethics approvals were obtained from the Hunter New England Human Research Ethics Committee (2021/ETH00319 and 2021/STE00569).

## Results

### Clinical record audit sample

A total of 175 participants were assessed for eligibility, 167 of which were eligible to participate. Two participants were excluded due to incomplete data. Complete data was obtained for 165 participants. Participant characteristics by CI screening status and are presented in Table 1. The mean age of participants was 81.89 years (SD = 7.71).

**Table 1:** Characteristics of sample by CI screening (n = 165)

		Screened for cognitive impairment	
		Yes (n = 57) n (%)	No (n = 108) n (%)
**Age category**	*65–74*	4 (13%)	28 (88%)
	*75–84*	20 (30%)	46 (70%)
	*85–94*	29 (48%)	31 (52%)
	*95+*	4 (57%)	3 (43%)
**Gender**	*Male*	27(33%)	55(67%)
	*Female*	30(36%)	53(64%)
**Relationship status**	*Married*	18 (31%)	40 (69%)
	*Widowed*	28 (44%)	35 (56%)
	*Divorced or separated*	9 (26%)	25 (74%)
	*Single*	2 (20%)	8 (80%)
**Number of admissions in the previous 12 months**	*0*	17 (49%)	18 (51%)
	*1*	23 (45%)	28 (55%)
	*2*	8 (22%)	28 (78%)
	*3 or more*	9 (21%)	34 (79%)
**Cognitive impairment**	*Dementia*	7 (41%)	10 (59%)
	*Mild CI*	4 (40%)	6(60%)
	*No*	46 (33%)	92 (67%)
**Number of co-morbid conditions**	*1*	0	2 (100%)
	*2*	2 (12%)	15 (88%)
	*3*	9 (33%)	18 (67%)
	*4 or more*	46 (39%)	73 (61%)
**Receiving palliative care**	*Yes*	1 (10%)	9 (90%)
	*No*	56 (36%)	99 (64%)
**Estimated ECOG**	*0*	0	2 (100%)
	*1*	1 (10%)	9 (90%)
	*2*	28 (32%)	59 (68%)
	*3*	27 (43%)	36 (57%)
	*4*	1 (33%)	2 (67%)

### Screening for CI

Of the 165 patients included, 34.5% (n = 57) had screening for CI documented in their medical record for their current admission. The most frequently used tools to conducted CI screening were the 4AT (n = 31, 54.4%), followed by the 6 Item Screener (n = 21, 36.8%) and the AMTS (n = 5, 8.7%). Factors associated with having screening completed are provided in Table 2. Those aged 85 years or older were 3.52 times more likely to have CI screening completed compared to those aged 84 or less (95% CI: 1.70, 7.30, p = 0.0007). Those who had two or more admissions in the preceding year were 0.24 times more likely to have CI screening completed compared to those with one or no previous admissions (95% CI: 0.11,0.50, p = 0.0001). Gender, functional status ECOG or number of comorbid conditions were not significantly associated with screening for CI.

**Table 2:** Factors associated with having screening for CI documented (n = 165)

		Crude			Adjusted	
**Characteristic**	**Response**	**OR** **(95% CI)**	**p value**	**OR** **(95% CI)**		**p value**
Gender	Male Female	Ref 1.15 (0.61, 2.19)	0.6639			
Age	< 84 ≥ 85	Ref 2.99 (1.54, 5.82)	0.0012	Ref 3.52 (1.70, 7.30)		0.0007
Number of admissions in last 12 months	1 or less 2 or more	Ref 0.32 (0.16, 0.62)	0.0009	Ref 0.24 (0.11, 0.50)		0.0001
ECOG status	0–1 2 3–4	Ref 4.75(0.58, 38.92) 7.37 (0.89, 60.95)	0.1469 0.0639	Ref		
Number of co-morbid conditions	2 or less 3 or more	Ref 5.14 (1.14, 23.09)	0.0328	Ref 8.49 (1.76, 40.9)		0.0077

### Rates of undocumented CI

Documentation of screening for CI by MoCA test result is provided in Table 3. Of patients who had screening documented, 75% (n = 43) were also identified as cognitively impaired following testing using the MoCA. Amongst patients who had no CI screening documented, 72% (n = 78) met criteria for CI using the MoCA.

**Table 3:** Presence of documentation for screening for CI in clinical record by MoCA test result (n = 165)

		MoCA result		Total N = 165
		Cognitive Impairment N = 121(73.3%)	No Cognitive Impairment N = 44 (26.7%)	
**Screening for CI documented in clinical record**	*Yes*	43 (75%)	14 (25%)	57 (34.5%)
	*No*	78 (72%)	30 (28%)	108 (65.5%)

### Qualitative sample

Interviews were conducted with a total of 20 healthcare providers. Participants included Junior Medical Officers (n = 10), Senior Resident Medical Officers (n = 8) and registered nurses of the Aged Care Service Emergency team (n = 2). Interview duration varied between 7 and 20 min.

### Perceived benefits to CI screening

Participants universally agreed that there were benefits to undertaking CI screening. These included ensuring patients received appropriate care, identifying delirium and/or dementia, establishing a baseline so that any deterioration during admission could be identified, providing information about capacity to make treatment decisions while in hospital, and providing critical information for care planning including the types of services patients may need following discharge.

*“You have a baseline you can see when there’s a deterioration from their baseline and also, you can identify issues earlier. So if there are any issues that need to be addressed, you can get processes and things in place to help them” (P16, JMO, < 1 year experience).*

*“In the short term, the benefit to screening is that you have patients that are higher risk of becoming, like delirious or agitated or aggressive, but then you also have the added benefit of, if you pick up a patient that you didn’t realise has a MoCA of 18, they probably should have an outpatient geriatrician follow-up, or have further assessment while they’re an inpatient… I think screening is always good” (P8, JMO, < 1 year experience).*

*“Screening would have higher sensitivity than just pure concern would. So, you might pick up patients that might not have the capacity that you think they do that’s actually happened to us last week. Shocking cognitive impairment in someone that we assumed was perfectly independent” (P5, SMRO, 3 years’ experience).*

### Current practices for CI screening

Commonly used tools for screening identified by participants were the 4AT, Mini Mental State Exam (MMSE), MoCA, and CAM, and several participants identified the suitability of the RUDAS for non-native English speakers. Healthcare providers reported that the conduct of screening was variable. While some participants reported screening every patient aged over 65 years, others reported that whether screening was completed depended largely on the symptoms of the presenting patient.

*“I think it really just depends on what they come in with, right? I wouldn’t do it - I don’t think- for every patient that I’ve seen, but if I start getting concerned that someone might be delirious, I’ll start the CAMs” (P16, JMO, 1 year experience).*

*“I think it would be dependent on the patient, definitely. Like, I’m not going to do a cognitive screen on someone who seems very with-it when I’m talking to them, and can fully recount a history and that kind of thing, but, if someone comes in, and they’re confused, especially if they’re confused and they’re over 50, over 60, then I would be performing it” (P19, JMO, 1 year experience)*.

### Barriers to CI screening

Lack of time and poor healthcare provider knowledge and awareness of CI screening were the two most commonly mentioned barriers to routine CI screening. In the emergency department, healthcare providers reported not having enough time to consistently undertake CI screening for patients aged 65 and older. Managing acute problems and bed block in stretched and busy emergency departments meant CI screening was not seen as a priority and was “*one of those things that just slips under the radar*” *(P3, JMO, 1 year experience).*

*“ I don’t think it is a lack of resources, I just think it is maybe a lack of sort of, it just - the hospital being an acute environment and you can get quite busy, it gets missed.” (P4, JMO, < 1 years experience).*

There was a clear lack of awareness among some participants about policies and expectations for screening. Participants reported they “*didn’t know about the policy, because I hadn’t done a medical term.” (P15, JMO, 1 year experience)* and even experienced staff reported they weren’t *“aware there was a policy for screening people over 65” (P18, SMRO, 3 years experience).*

Despite this, several participants acknowledged that CI screening could be completed relatively quickly using short tools, and that “*it’s pretty easy, so there shouldn’t really be a reason not to do it*” (P2, JMO, *< 1 years experience)*. Screening was more often thought to occur on wards where there was sufficient time and often a dedicated healthcare provider who took responsibility for completing screening, although some participants also mentioned that screening was routinely conducted in the emergency department.“*Most of the patients up here on this ward tend to receive them, because I think the staff-to-patient ratio up here is more appropriate” (P8, JMO, 1 year experience). “We start the process here in ED, and then the idea is if they’re triggering any red flags that we then pop them onto a pathway that forms part of our handover to the ward and then they continue to monitor their cognition.” (P12, Nurse, 2 years experience).*

While almost all healthcare providers were aware of the various CI screening tools, almost one quarter were unaware of hospital policy for CI screening to occur for overnight admitted patients aged 65years and over. Participants reported that lack of knowledge of policies and differences in practice across the hospital contributed to low rates of CI screening

*“I wasn’t aware there was a policy for screening people over 65” (P18, SMRO, 3 years experience)*.

*“If it’s new staff to the department, they haven’t had that education. So, they’ve come from another team who don’t routinely screen for cognition and delirium. They may not know that we do that as par for the course here. (P12, Nurse, 2 years experience).*

Participants sometimes reported confusion about which CI screening tool was appropriate to use in different situations, and how to appropriately administer them. There was also some confusion about whose responsibility it was to complete screening, with some participants seeing it as a responsibility or nurses or allied health providers.

*“From doing MoCAs in the past, what I struggled with was knowing when a patient will get the answer, like how much time you give them, and how patient you are with them being - with them answering and how much is okay. Also, how many hints you can give before it not counting as a point?” (P15, JMO, 1 year experience)*.

“*I don’t think we’ve specifically been told to screen… I’ve seen allied health get involved by maybe the nurses taking initiative and getting OTs involved… I haven’t done a lot of screening myself” (P15, JMO, 1 year experience).*

*“My understanding, I think, was that if the patient is for discharge, then they’ll be seen by our set nurse, and they do it. But I guess in terms of patients being admitted, I have never really thought about who’s doing it. I guess that should fall to me.” (*P2, JMO, *< 1 years experience).*

*“For me, it was just mainly having an unclear idea of when to do it and if I’m to do it.”* (P2, JMO, *< 1 years experience)*.

## Discussion

The routine and accurate detection of CI is key to improving quality of healthcare provided to older adults in hospitals. This mixed methods study aimed to understand completion of CI screening and care planning in the clinical record of hospital inpatients aged 65 years and older, as well as understand healthcare provider perspectives on practices and barriers to CI screening.

Overall, only 34.5% of inpatients had screening for CI documented in their clinical records, despite a policy directive requiring screening for all overnight admitted patients aged ≥ 65 years [16–19]. Failure to detect CI has serious medical and legal implications. Healthcare providers may wrongly assume capacity to understand complex risk–benefits of treatments, and CI is associated with increased hospital-acquired complications, increased length of stay and unplanned readmissions [8, 9, 12, 40–42]. However, identification of CI through screening can prompt the provision of “dementia-friendly” hospital care [6, 14, 43] including non-pharmacological interventions to prevent delirium, avoiding medications with cognitive side-effects, improved communication with patients, inclusion of caregivers and/or SDM in decision making, early medical and psychosocial interventions to patients with CI and their families [44, 45] and supported adequate discharge management [6, 15, 43]. Previous research has found that low rates of screening may result from the lack of a single endorsed validated tool for cognitive screening [46, 47], poor staff awareness of hospital policies [4] and understanding of the importance of screening in improving health outcomes, insufficient time to undertake screening, and cognitive assessment being considered a lower priority compared to physical care. Similar barriers were identified by healthcare professionals in our study, who identified lack of awareness of CI screening policies, lack of knowledge about which screening tool to use as key barriers to routine implementation of CI screening. These findings highlight the need for ongoing training for clinical staff in the use of available short screening tools.

The prevalence of undocumented CI as assessed by the MoCA was alarmingly high at 72%. While we are, to the best of our knowledge, the first to describe rates of undocumented CI by comparing CI screening tests at admission with a validated CI screening test,, this rate of undocumented CI is higher than previously described in literature both nationally and internationally. A prospective observational cohort study examining CI amongst Australians aged over 70 years admitted to four hospitals in Queensland reported 29.4% had CI and 20.1% dementia [5]. Of those who were diagnosed with dementia, more than half did not have documentation of this in their clinical record [5]. International studies with varying methodology show a broad range of rates of undocumented CI when compared to medical records between 46 and 62% [48, 49] and undocumented dementia between 46 and 64% [11, 20, 50, 51] in admitted older hospital inpatients. These differences likely reflect variability in demographics of patient populations, the clinical setting, and the screening and diagnostic assessment methods used to determine CI. While some studies used MMSE, in our study the MoCA was used given its greater sensitivity at detecting MCI [30, 52–54], early vascular CI [55] and CI due to Parkinson’s disease [56]. Studies also administered screening at different timepoints following admission, which is important given screening is best done once acute illness has resolved. Our study population was older than other studies and included patients from institutionalized care, where rates of CI are higher [7, 57, 58]. Nevertheless, our data suggests there are significant numbers of older people who have CI that are not recognised, and therefore may not be receiving appropriate patient-centred care.

### Strengths and limitations

A strength of this study is the use of the MoCA to screen for CI rather than the MMSE, as the MoCA has been shown to be more sensitive to milder forms of CI, vascular CI and CI related to Parkinson’s disease. The face-to-face administration of the MoCA by a trained geriatric trainee to a large sample of patients, and systematic sampling of patients, are also strengths of this study.

Several limitations should be noted. Firstly, the study was conducted in a single centre and therefore findings may not be generalisable to other healthcare settings. Secondly, screening for CI was limited to a single MoCA assessment but vital information gained from collateral history of subjective memory complaints from caregivers was not incorporated. The MoCA is designed as a stand-alone screening tool, but further testing is generally required to make a formal diagnosis of CI. Thirdly, this is a prospective clinical record audit hence some information about cognitive screening performed at the bedside but not documented in the clinical record may have been missed. Finally, interviews were limited to medical officers and ASET aged care nursing staff, but excluded other relevant health professionals such as occupational therapists and medical ward nursing staff. The small number of nurses included is a limitation given nurses often have a key role to play in screening for CI during admission.

## Conclusion

Medical admissions offer a timely opportunity to identify CI to ensure high-quality care is provided and strategies are implemented to minimise adverse events associated with CI. Despite policy recommendations for routine screening of hospitalised inpatients aged over 65 years, many patients are not screened. With the rapidly growing ageing population, further research is warranted to identify feasible and effective strategies to increase implementation of CI screening, to provide quality patient-centred care.

### Electronic supplementary material

Below is the link to the electronic supplementary material.

Supplementary Material 1

## Data Availability

The datasets used and/or analysed during the current study will be available from the corresponding author on reasonable request.

## Declarations

New England Human Research Ethics Committee (2021/ETH00319 and 2021/STE00569). Healthcare providers participating in interviews provided informed consent for participation. A waiver of consent was provided by the Hunter New England Human Research Ethics Committee for the conduct of the clinical audit.

## List of abbreviations

AMTS: Abbreviate Mental Test Score
CI: Cognitive impairment
ECOG: Eastern Cooperative Oncology Group
MMSE: Mini Mental State Exam
MoCA: Montreal Cognitive Assessment
RUDAS: Rowland Universal Dementia Scale
