# Supplementary material for: Documentation of cognitive impairment screening amongst older hospitalised Australians: a prospective clinical record audit
Source: BMC Geriatr. 2023 Oct 18;23:672. doi: 10.1186/s12877-023-04394-z (PMC10583351; doi:10.1186/s12877-023-04394-z)
Supplement: Supplementary file 1 — Supplementary Material 1 [file 12877_2023_4394_MOESM1_ESM.docx]

**Appendix A**

Interview Guide

V1, 24/02/2021

Thank you so much for taking the time to talk to me today. As discussed, this interview is expected to take approximately 30minutes of your time. Please let me know if you want to stop at any time or if there are any questions you would prefer not to answer. I am starting the tape recorder now [if consent provided].

I would like to talk to you today about screening for cognitive impairment. I’d like you to answer these questions by reflecting on your clinical experience in providing care to older patients. The information you provide today may assist in the development of new strategies for improving the implementation of cognitive screening at Hunter New England Hospitals.

**First, I’d like to ask a few questions about you.**

1. What is your current position?
2. How many years have you been in your current position?
3. What are you qualifications?

**Great, thank you. Now I’d like to ask some questions about screening for cognitive impairment.**

1. Are you aware of any hospital policies and procedures relating to screening for cognitive impairment for older adults?
2. Can you tell me about screening tools for cognitive impairment that are used with older patients being admitted to hospital?
   - Are you familiar with any screening tools?
   - Is there a particular screening tool you use?
   - Is there a particular screening tool that your colleagues use?
   - Does the type of tool used vary (e.g. by patient)? If so, why?

AMTS

1. On average, how often is screening for cognitive impairment done?
   - Every admission, majority of admissions, minority of admissions, occasional?
   - What proportion of patients aged 65 or older being admitted to hospital from the emergency department would receive screening for cognitive impairment?
2. What influences whether someone receives screening for cognitive impairment?
   - Patient related factors, e.g. age, health condition
   - Provider related factors, e.g. time, training
   - Other factors?
3. What do you perceive are the benefits of screening for cognitive impairment in patients getting admitted to hospital?
4. What are barriers to screening older patients for cognitive impairment?
5. What do you think might help to improve rates of screening?
   - Training and education? For all staff or specific staff?
   - Systems and processes for ensuring that screening takes place?
   - Additional staff resources?
   - Other?

These were all the questions I wanted to ask you today. Just to let you know, I am turning the tape recorder off now. I really appreciate you taking the time to speak with me today. The information you have provided is really helpful.
